# Supplementary material for: A Bayesian Account of Generalist and Specialist Formation Under the Active Inference Framework
Source: Front Artif Intell. 2020 Sep 3;3:69. doi: 10.3389/frai.2020.00069 (PMC7861269; doi:10.3389/frai.2020.00069)
Supplement: Supplementary file 1 [file Data_Sheet_1.pdf]

Supplementary Document

A Bayesian account of generalist and specialist formation under the Active Inference framework

Appendix A: Bayesian model comparison

A.1 Derivations of Bayesian Model Comparison

We start off with the following approximate equalities of the approximate posterior of a set of parameters (50):

$$\delta_Q F = 0$$

$$\Rightarrow Q(\theta) \approx P(\theta|\tilde{o}) \qquad \text{(Equation A1)}$$

$$\Rightarrow -F[P(\theta)] \approx \ln P(y)$$

$\theta = (\theta_1, \theta_2, \dots)$ is used here to denote any arbitrary set of parameters, and $\delta_Q F = 0$ means the variation of the free energy with respect to the approximate posterior is zero (i.e. a stationary point of the free energy). For the purpose of policy learning as discussed in this paper, it would be identical to substitute the tuple of concentration parameters, e, in lieu of θ below.

In order to perform Bayesian model comparison (BMC), we define our two models: a *full* model (in this case, the model the agent used in the previous day), and a *reduced* model (model constructed during BMC which the agent compares against the full model). We define the probabilities under the

two models with the P_F and P_R, respectively. Crucially, we make a key assumption, that the likelihood of observing the outcomes is equally likely under both models:

$$P_F(\tilde{o}\,|\theta) = P_R(\tilde{o}|\theta) \tag{A2}$$

We begin by writing out Bayes rule to both the full and reduced models:

$$\frac{P_R(\theta|\tilde{o})\,P_R(\tilde{o})}{P_F(\theta|\tilde{o})\,P_F(\tilde{o})} = \frac{P_R(\tilde{o}|\theta)\,P_R(\theta)}{P_F(\tilde{o}|\theta)\,P_F(\theta)}$$

Using the equality in Equation A2 to cancel the likelihood terms, and rearranging, we arrive at the following equality:

$$P_R(\theta|\tilde{o}) = \frac{P_R(\theta)}{P_F(\theta)}\,\frac{P_F(\tilde{o})}{P_R(\tilde{o})}\,P_F(\theta|\tilde{o}) \tag{A3}$$

Integrating both sides:

$$\int P_R(\theta|\tilde{o})\,d\theta = 1 = \int \frac{P_R(\theta)}{P_F(\theta)}\,\frac{P_F(\tilde{o})}{P_R(\tilde{o})}\,P_F(\theta|\tilde{o})\;d\theta$$

$$1 = \frac{P_F(\tilde{o})}{P_R(\tilde{o})}\;\int P_F(\theta|\tilde{o})\,\frac{P_R(\theta)}{P_F(\theta)}\;d\theta$$

$$P_R(\tilde{o}) = P_F(\tilde{o})\int P_F(\theta|\tilde{o})\,\frac{P_R(\theta)}{P_F(\theta)}\,d\theta \tag{A4}$$

$$P_R(\tilde{o}) \approx P_F(\tilde{o})\int Q_F(\theta)\,\frac{P_R(\theta)}{P_F(\theta)}\,d\theta \qquad \text{[Substituting in A1]}$$

$$\ln P_R(\tilde{o}) \approx \ln\int Q_F(\theta)\frac{P_R(\theta)}{P_F(\theta)}\,d\theta + \ln P_F(\tilde{o}) \qquad \text{[Taking the logarithm]}$$

$$= \ln E_{Q_F}\left[\frac{P_R(\theta)}{P_F(\theta)}\right] + \ln P_F(\tilde{o})$$

$$\ln P_R(\tilde{o}) \approx -F[P_R(\theta)] \approx \ln E_{Q_F}\left[\frac{P_R(\theta)}{P_F(\theta)}\right] - F[P_F(\theta)] \quad \text{[Substituting in A1]} \tag{A5}$$

Equation A5 tells us that the model evidence of any reduced model can be evaluated given the prior of the reduced and full models, and the evidence of the full model. Applying the above knowledge to the e concentration parameters defined previously, we have the following:

$P_F(\theta) = Dir(e_F)$ Prior of the full model

$P_R(\theta) = Dir(e_R)$ Prior of the full model

$Q_F(\theta) = Dir(\boldsymbol{e_F})$ Prior of the full model

$Q_F(\theta) = Dir(\boldsymbol{e_R})$ Prior of the full model

In order to compare relative model evidence, we look at the log ratio of the reduced and full model evidence, which is the same as the difference in their free energy (free energy of the full model minus the reduced):

$$\Delta F = \ln \frac{P_R(\tilde{o})}{P_F\,(\tilde{o})} = \ln P_R(\tilde{o}) - \ln P_F(\tilde{o})$$

In the discrete case, the above can simply be re-written with Beta functions $\mathrm{B}(\cdot)$ (50):

$$\Delta F = \ln \mathrm{B}(e_F) - \ln \mathrm{B}(e_R) - \ln \mathrm{B}(\boldsymbol{e_F}) + \ln \mathrm{B}(\boldsymbol{e_F} + e_R - e_F) \tag{A6}$$

We can apply the above to any reduced model to evaluate its evidence relative to the full model. Intuitively, the higher ΔF is, the more evidence the reduced model has. We can evaluate ΔF for an arbitrarily large number of reduced models.

In the case of *Bayesian model selection*, the reduced model with the highest model evidence is selected as the optimal model. That is to say, given a vector of the relative free energy for each reduced model, $\Delta \mathrm{F}$, we pick the e_R which gives $\max(\Delta \mathrm{F})$. However, since we are interested in *Bayesian model averaging*, we need to compute the probability of each reduced model within the entire reduced model space we defined:

$$\boldsymbol{m} = \sigma(\Delta \mathrm{F}) \quad \text{(A7)}$$

where $\boldsymbol{m}_i = Q(m = i)$ is the posterior probability of each reduced model and σ is the softmax function, $\sigma(x) = \frac{\exp(x)}{\sum \exp(x)}$, which squashes the set of values in vector $\Delta \mathrm{F}$ into a range that is between [0, 1] and sums to 1 (i.e. forms a probability distribution). After the probability of each reduced model is computed, we simply take a weighted sum of each reduced model parameters, weighted by their probability, to get the final, Bayesian model averaged parameters:

$$\boldsymbol{e}_{i,BMA} = \boldsymbol{m} \cdot \boldsymbol{e}_{i,R} \quad \text{(A8)}$$

where $\boldsymbol{e}_{i,R}$ is a vector of the *i-th* concentration parameters for each reduced model, and $\boldsymbol{e}_{i,BMA}$ is the *i-th* Bayesian model averaged concentration parameter over all reduced models.

A.2 Example application of Bayesian model comparison to maze task

Taking our "two-step" maze task for example, let us imagine an agent that repeatedly pursues policy 1 (Fig 3B) throughout the day. At the end of the day, having completed 8 trials, its *e* parameter for policy 1 has increased from a prior concentration of 1 to a posterior concentration of 9 (Fig A1a). The agent then performs model comparison ("sleep"), where it entertains possible combinations of reduced models for prior *e* parameters (Fig A1b) and computes the model evidence for each reduced model using the derivations shown in Appendix section A.1 (the resulting model evidence is shown in Fig A1c). Specifically, it tests different initial parameters (Fig A1b) in lieu of the true prior parameters used (Fig A1a, left) to see whether these provide better explanations for the observed data (Fig A1a, right).

The reduced models (Fig A1b) are constructed via strengthening certain policies (increasing their *e* parameters, akin to synaptic strengthening) and weakening others (decreasing *e* parameters, akin to synaptic pruning). The point is to construct many reduced models such that the model space is more likely to contain many good models, and a search through them will pick up those good models (hypothetically, the reduced model space can be arbitrarily large). In our case, we increment the *e* parameter of the to-be-strengthened policies by 8 and divide the *e* of to-be-weakened policies by 2 or 4. The reason for this numerical manipulation is twofold. Firstly, it is more neurobiologically plausible to weaken policies (e.g. via weakening synaptic connections, or in our case, decreasing the *e* parameter by dividing) over time as supposed to "deleting" policies altogether when they are not used. In practice, when the probability of a policy becomes sufficiently small, we can associate this with the pruning of the synapses. Secondly, it is beneficial to construct a large reduced model space, which helps Bayesian model reduction to find a more optimal reduced model. In total, each time model reduction occurs, it iterates through all combinations of reduced policies (since we have 7

policies and we can either strengthen or weaken each one, we have $2^7 = 128$ combinations) with the two levels of pruning discussed above for a total of 256 reduced models to average over. Figure A1b, left is an example of a reduced model, in which policy 1 is strengthened (more probable), and all other policies weakened. This is the reduced model with the best model evidence, since it corresponds with the agent's action during the day (Fig A1a, right).

Now that the probability of each model within the reduced model space is computed (Equation A7, visualized in Fig A1c), we perform Bayesian model averaging get a weighted sum over all the models (Equation A8). The resulting prior (e_{BMA}) is the optimal set of prior parameters that the agent could have started the previous day with, given the reduced models considered. Finally, the amount of learning (i.e. increases in *e* for policy 1 by 8) is added to this "optimised prior" to get the most optimal posterior *e* concentration, (Fig A1d, right), which is used as the prior concentration for the subsequent day. This is the posterior that the mouse would have reached, had it started with the best prior. This process repeats after each day of training, where the agent continually optimises its parameters to inform better future policy selection.

A.3 Relevant Notations

The Markov Decision Process is specified using the following matrices

$\boldsymbol{A_{ij}} = P(o_\tau = i \mid s_\tau = j)$	state-outcome mapping
$\boldsymbol{B(u)_{ij}} = P(s_{\tau+1} = i \mid s_\tau = j, u = \pi(\tau))$	state-state transition
$\boldsymbol{C_{\tau,i}} = P(o_\tau = i)$	outcome preference
$\boldsymbol{D_i} = P(s_1 = i)$	belief about initial states
$\boldsymbol{E_i} = P(\pi = \mathrm{i} \mid \mathrm{E})$	independent policy

We define the (free energy independent) prior and posterior distributions over the parameter E (which determines the policy space) as:

$$P(E) = Dir(e)$$

$$Q(E) = Dir(\boldsymbol{e})$$

$$\boldsymbol{e} = e + \boldsymbol{\pi}$$

Where $e = (e_1, \ldots, e_k)$ are the prior concentration parameters, $\boldsymbol{\pi} = (\boldsymbol{\pi_1}, \boldsymbol{\pi_2}, \ldots, \boldsymbol{\pi_k})$ is the posterior probability that an agent observes itself pursuing each of the (k) policies, and $\boldsymbol{e}$ is the posterior concentration parameter. We therefore have the prior (free energy independent) expectation about policies:

$$\hat{E} = \mathbb{E}_{P(E)}[\ln P(\pi|E)]$$

Finally, policy inference is:

$$\boldsymbol{\pi} = \sigma(\boldsymbol{\hat{E}} - \boldsymbol{F} - \gamma \cdot \boldsymbol{G})$$

Where $\boldsymbol{F}$ is the free energy for each policy based on past time points and $\boldsymbol{G}$ is the expected free energy for future time points (modulated by a precision term γ). At the end of the trial, the prior $\widehat{\boldsymbol{E}}$ is updated to the posterior $\mathbb{E}_{Q(E)}[\ln P(\pi|E)]$, which is used as the new prior for the next trial.

Appendix B: Software note

The simulation is constructed using MATLAB (https://www.mathworks.com/products/matlab.html) and the SPM12 software package (https://www.fil.ion.ucl.ac.uk/spm/). Specifically, the DEM toolbox in SPM12 is used to run the Active Inference simulations. All of the scripts used specifically for this experiment can be found on GitHub (https://github.com/im-ant/ActiveInference_PolicyLearning).

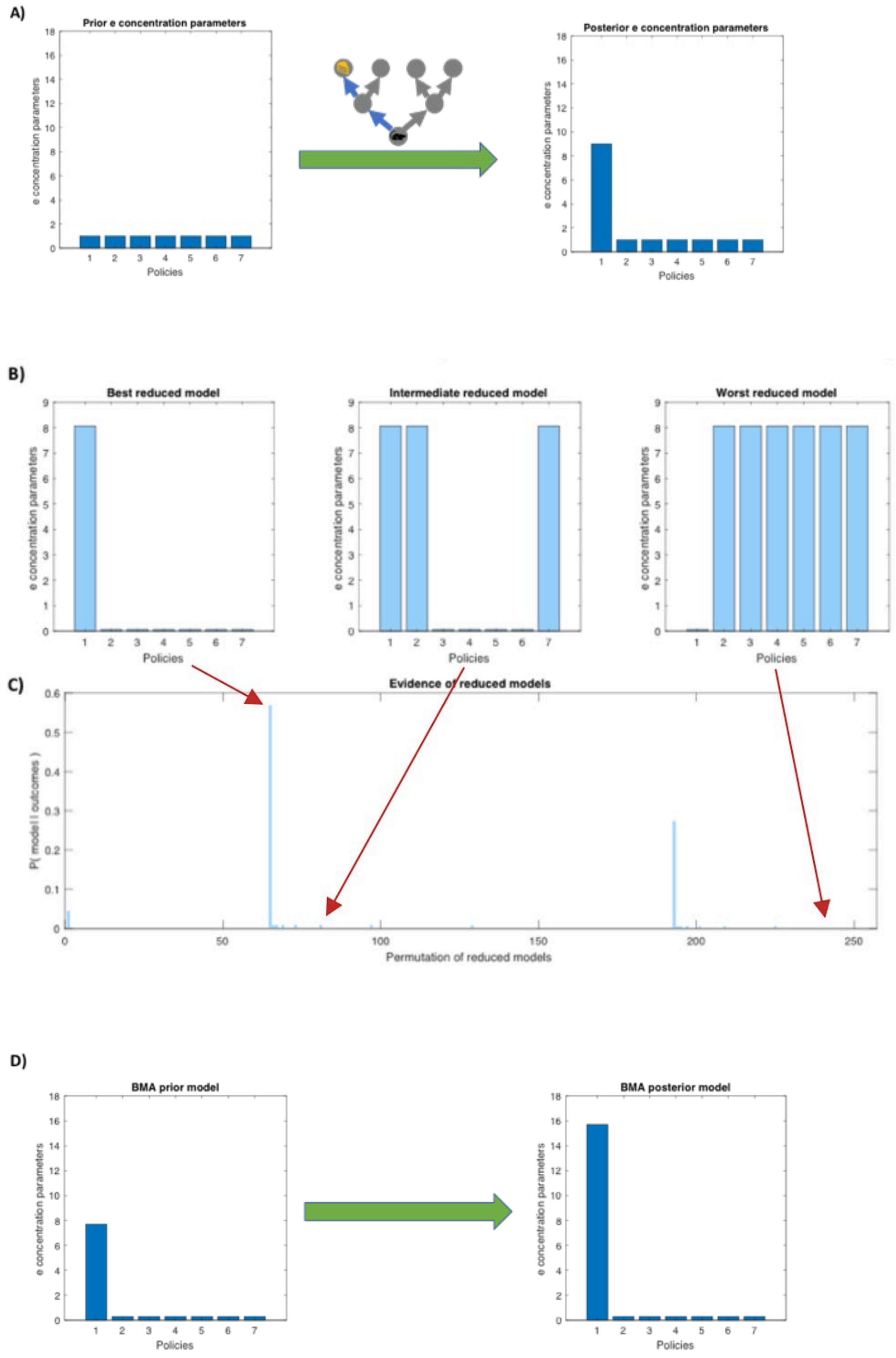

Figure A1: Bayesian Model Averaging (BMA). (a) The effect of training on the *e* concentration parameters. The agent pursues policy 1 eight times during the day, and subsequently the *e* parameter for its policy 1 incremented from 1 to 9. **(b)** Example of reduced models. In our case, reduced models are

prior *e* concentration parameters that try to better the posterior e concentration observed at the end of the previous day (i.e. part A, right). **(c)** Examples of model evidence. We see the reduced (prior) model increased *e* concentration for policy 1, and decreased concentration for all other policies received the highest model evidence (i.e. it is the best reduced model), whereas models that do the opposite have low model evidence. **(d)** Updating the prior *e* concentration after BMA. The agent first computes the BMA-ed prior *e* concentration (left bar graph), then adds on the amount of learning done during the day to computed the BMA-ed *posterior* e concentration, which is used as the prior for the next day.
